# A Model for Predictive Maintenance Based on Asset Administration Shell

**DOI:** 10.3390/s20216028

**Published:** 2020-10-23

**Authors:** Salvatore Cavalieri, Marco Giuseppe Salafia

**Affiliations:** Department of Electrical Electronic and Computer Engineering, University of Catania, 95125 Catania, Italy; salvatore.cavalieri@unict.it; Tel.: +39-095-738-2362

**Keywords:** predictive maintenance, industry 4.0, digital twin, asset administration shell, CPS, IIoT

## Abstract

Maintenance is one of the most important aspects in industrial and production environments. Predictive maintenance is an approach that aims to schedule maintenance tasks based on historical data in order to avoid machine failures and reduce the costs due to unnecessary maintenance actions. Approaches for the implementation of a maintenance solution often differ depending on the kind of data to be analyzed and on the techniques and models adopted for the failure forecasts and for maintenance decision-making. Nowadays, Industry 4.0 introduces a flexible and adaptable manufacturing concept to satisfy a market requiring an increasing demand for customization. The adoption of vendor-specific solutions for predictive maintenance and the heterogeneity of technologies adopted in the brownfield for the condition monitoring of machinery reduce the flexibility and interoperability required by Industry 4.0. In this paper a novel approach for the definition of a generic and technology-independent model for predictive maintenance is presented. Such model leverages on the concept of the Reference Architecture Model for Industry (RAMI) 4.0 Asset Administration Shell, as a means to achieve interoperability between different devices and to implement generic functionalities for predictive maintenance.

## 1. Introduction

Maintenance is of paramount importance for industrial or production plants. The main aim of maintenance is maximizing the production of the plant whilst reducing the costs as much as possible. Maintenance costs are some of the main components of the total cost of a production facility as they can represent between 15 and 60% of the cost of goods produced [1]. The strategy adopted for maintenance management has a huge impact on the overall cost of the plant, thus, there are different maintenance strategies being used to maintain the efficiency [2]. Surveys demonstrated that one-third of all maintenance costs are wasted because of unnecessary or improperly carried out maintenance [1]. The approach referred to as predictive maintenance (PdM), consists of scheduling maintenance actions when either a deterioration or a degradation in the performances of the machine is detected. A comprehensive maintenance management program based on PdM optimizes the availability of the equipment and greatly reduces the cost of maintenance in general. Even product quality, productivity, and profitability of the production plant are improved [1].

Nowadays, we are facing a new industrial revolution, often referred as Industry 4.0, introducing the application of modern information and communication technologies (ICT) concepts, like the Internet of Things (IoT), in industrial contexts to create more flexible products and services leading to new business models and added value [3,4]. Furthermore, Industry 4.0 introduces a flexible and adaptable manufacturing concept to satisfy a market requiring an increasing demand for customization [5]. In this context, maintenance is of paramount importance since avoiding failures, and thus, money loss, is a key requirement in a challenging market requiring high efficiency and availability. For these reasons, maintenance in Industry 4.0 is considered value creator [6]. Furthermore, the process of digitalization and the new forms of cooperation between companies introduced in Industry 4.0 lead to the definition of new business models introducing new services and solutions. Value can be generated by analyzing data and using them as a source for competitive advantage [7]. Maintenance data may be used as a value proposition for a new data-driven business model. For instance, as stated in [8] and [9], the interconnection across supply chains by digital means enables new potentials, from process optimization to new business models, such as “hardware as a service”. Analytics data, like data used for and generated by the maintenance process, may also be used by partners of the value chain (i.e., machinery suppliers) to introduce new services for customers or to improve their products.

It is a matter of fact that the literature presents a lot of different PdM solutions involving different technologies and approaches, from the gathering of data to the prognostics of failures. Furthermore, in the context of industrial environment and the fourth industrial revolution, lots of different technologies and protocols are adopted from the brownfield area (i.e., sensors, fieldbuses) to the IT area. The adoption of vendor-specific solutions for PdM reduces the flexibility and interoperability required by Industry 4.0. Therefore, it is hard to define a PdM solution that can adapt itself to the variation in the configuration of the original production, setting new constraints to the flexibility in the smart factory. New mechanisms are required for the horizontal integration hiding implementation details and guaranteeing a communication channel between devices, regardless of both their adopted manufacturers and technologies. In this manner, a device can be easily replaced with an equivalent one providing the same functionalities (i.e., production functionalities and PdM functionalities). Another important aspect, is the transmission of heterogeneous data coming from devices to the enterprise levels; such data must be presented in a uniform manner to guarantee a vertical integration between the several components of the PdM solution. To cope with this issue, the fourth industrial revolution introduced the concept of cyber-physical systems (CPS) and digital twins (DT) to create a uniform digital representation of assets in the production value chain. This digitalization process allowed the definition of further novel approaches to PdM, like those discussed in [10,11]. However, all of these solutions often refer to specific scenarios and do not face the problem of reconfiguration of production and do not discuss the disruptive impact it has on the PdM solution, which needs to be re-engineered to be adapted to the new changings in the plant. Furthermore, if PdM solutions can be described using generic steps and functionalities independent from the actual implementation, they can be easily adapted to changings in the production configuration.

In this paper a logical model for PdM will be presented highlighting all the “common factors” between the PdM solutions in the literature, generalizing all the aspects that a PdM solution implementation should cope with and representing them in suited logical blocks collecting all the relevant functionalities. The presented PdM model is based on the so-called Asset Administration Shell (AAS) presented in the Reference Architecture Model for Industry 4.0 (RAMI4.0) [12], which realizes the concept of DT. The proposed approach leverages on AASs because they create a standardized abstraction layer above assets using different technologies that cannot interoperate each other. Using the same AAS model [13,14] to expose different data modules by means of a standardized external interface makes the devices seamlessly interoperable [15], solving the aforementioned issue concerning the massive heterogeneity of technologies and information models adopted in the industrial environment.

The paper is structured as follows. In Section 2 the related work is discussed. In Section 3, an overview of the main concepts about PdM and AAS will be provided to the reader as a background for the paper comprehension. Section 4 presents the AAS-based PdM model proposed by this work. In Section 5 a cloud-based PdM solution based on the PdM model is presented as a use case. In Section 6, conclusions and outlook are discussed.

## 2. Related Studies

An approach for the definition of a PdM program that satisfies the requirements of flexibility and interoperability demanded by Industry 4.0 must address two main objectives: (1) defining generic functionalities for the description of a technology-independent PdM solution and (2) hiding the heterogeneity and complexity of the operational technology (OT) level. Both the objectives are often addressed either separately or partially (see [16,17,18]) but, to the best of our knowledge, there is no solution facing both together. Such objectives were already known in 2007, where Groba et al. [19] analyzed the aspect of PdM and the challenges to face considering such an approach. The aim of their proposal is the definition of a PdM framework integrating the diversity of different PdM techniques. Their framework copes only with (1), but they identified that one of the biggest challenges consists in describing the shop floor equipment and corresponding condition indicators in a uniform manner, hence (2). Traini et al. [20] presented a basic framework for PdM of a generic manufacturing tool but it is based on Machine Learning (ML) techniques only, and does not consider the flexibility required for a smart factory. In [21] a framework for a flexible maintenance platform is proposed enabling modularization of related functions, but it also relies on AI techniques for the failure predictions, and the integration of devices and data is addressed partially. A similar approach to our proposal is discussed in [22] where a framework based on blocks providing a comprehensive representation of key components of a generic PdM solution is discussed. The objective of this framework is a reduction in the complexity behind PdM programs’ management, but it does not consider the issues related to the interoperability of devices and data required by Industry 4.0.

The requirements for (2) consist essentially of issues faced nowadays by CPS and DT in the context of Industry 4.0. As mentioned in Section 3.2, AAS implements the concept of DT in RAMI4.0 and it is confirmed by Moyne et al. [23] who say that RAMI4.0 AAS satisfies some of the main requirements for DT proposed in their work. In particular, in the area of production automation, the intention of using AAS for future implementation of PdM solutions is confirmed in [24], where an appropriate infrastructure, consisting of components with uniform interfaces is of utmost importance for condition monitoring and PdM (see [25]). Our proposal leverage on the adoption of AAS to addresses (2) and defines modules of generic functionalities for PdM (where some of them are implemented in specific AAS submodels to address (1). Authors already adopted the AAS to abstract the complexity of plant configuration [26], but this proposal presents an approach for the representation of a PdM solution in terms of generic and technology-independent functionalities where AASs are used mainly in brownfield to achieve interoperability among devices.

## 3. Background

In general, a PdM program is strictly dependent on the data and technologies adopted, and eventual changings in these last, or new additions in the program’s configuration, may lead to a re-engineering process of the PdM solution. As mentioned in the introduction, this is not something desired in a competitive market where the availability of the production is of utmost importance. The definition of steps and functionalities for a generic PdM solution eases its management but, as previously discussed, since it is strictly dependent on the underlying technologies, an abstraction mechanism (i.e., AAS) is required to masquerade all the implementation details and harmonize data and operations in a uniform manner. This section provides the reader with an overview of PdM approaches and of the AAS to improve the paper’s comprehension. The work presented in this paper is based on the analysis of different approaches presented in literature for PdM in order to define functionalities for a generic PdM solution, whilst leverage on RAMI 4.0 AAS to hide the heterogeneity of underlying technologies adopted. Therefore, the model proposed here can be used to describe a PdM solution abstracting its description from its implementation, so that eventual changings in production configurations do not affect, in a disruptive way, the PdM program.

### 3.1. Overview on Predictive Maintenance

PdM is a maintenance approach that tries to prevent catastrophic failure of scheduling maintenance operations and detects the type of failure on the basis of the current condition of the machine. PdM is also referred to in the literature as condition-based maintenance (CBM) since it uses actual operating conditions of the equipment to predict the future state of the machine, using a model defined on the basis of historical data [2]. The foundation of predictive maintenance is the condition monitoring (CM) process [16], where sensors are applied in machinery to continuously monitor signals, or other appropriate indicators, to assess the health of the equipment [27]. To give some examples, the ac component of the output of pressure sensor (e.g., noise) can be used to detect blockages in the pipe of a plant [28], and vibration analysis gives an indication about the reliability and safety of a rotating machine [29].

Since PdM is a broad area, with lots of approaches and techniques involved, in this section we will point out the main parts composing a generic PdM approach. A CBM program can be divided in three main parts: data acquisition, data processing, and maintenance decision-making [30].

#### 3.1.1. Data Acquisition

Data acquisition is the process of collecting data from the physical assets, which are the subject of the predictive maintenance program. Sensors are the primary source of information here and the kinds of data collected vary case by case, depending on the machine to be maintained and hence on the sensor used. Hashemian and Bean [28] identify three major categories for predictive maintenance depending on both the kind of information acquired and the source adopted. Most of the time, old equipment requires the additions of new sensors [31] and the need for new tools for monitoring has made the adoption of PdM quite expensive in the past [6,27]. Nowadays, new technologies in the field of data acquisitions, like the IoT and sensor technology, make the adoption of PdM solutions more accessible [28,31,32]. Anyway, often the brownfield already contains smart equipment providing more information than just basic data over intelligent communication protocols, but systems from different vendors may run in different parts of the production process and a way to allow them to understand the information exchanged must be provided (e.g., gateways).

#### 3.1.2. Data Processing

After the data has been collected, the next step involves data cleansing in order to remove, for instance, sensor errors that may affect the data analysis. Often data are subjected to a pre-processing step where the volume of data is reduced (i.e., aggregation) to pass only the selected and extracted indicators (i.e., feature extraction) [31] to the forecasting and/or decision-making algorithms. The techniques adopted to process and analyze data mainly depend on both the types of data collected and the algorithms used to reveal the condition of the machine. In [30], the kind of data to be analyzed and the techniques for data analysis and signal process are deeply described.

All features extracted in this step are indicators of the health condition of the equipment or can be used for an estimation about the remaining operational time until a failure occurs. The reader can find more details about the most common techniques correlating such indices to the machine’s condition in [1].

#### 3.1.3. Maintenance Decision-Making

The techniques adopted for maintenance decision-making in CBM are classified in diagnostics and prognostics. The former deals with finding the source of a fault, whilst the latter deals with estimating when a failure may occur in future. It follows that prognostics are better than diagnostics because in the first case prevention of the failure is attempted, whilst in the second case the failure has already occurred. Of course, prognostics cannot prevent all faults; for this reason, diagnostics techniques are often used as a complementary support for prognostics. Furthermore, diagnostic results can be used as feedback to improve the accuracy of prognostic solutions [30].

Decision strategies for machine fault diagnostics are usually procedures that create correlations between data and/or features collected from measurements of features of the faults, and thus create a sort of classification over measurement data. This mapping process is also called pattern recognition. Usually, diagnostic solutions adopt statistical approaches or AI over the information collected with condition monitoring, but model-based solutions exist too. Some statistical approaches are presented in [33,34,35]. AI solutions, instead, have shown better performance compared to conventional approaches to diagnostics [30]. Common AI techniques instead are shown in [36,37,38]. Model-based solutions instead use physics or mathematical modelling to simulate the behavior of the monitored machine [39].

Most of the strategies adopted for prognostics, instead, involve the prediction of the so-called remaining useful lifetime (RUL), which indicates how much time is left until a failure occurs. Other solutions usually adopted in high-risk environment (where a failure is catastrophic) consist in calculating the probability that a failure occurs between two inspections of the plant. By the way, approaches considering RUL estimation constitute the majority of the literature. There are three families of RUL estimation models: similarity model, degenerate model, and survival model [40,41]. Of particular interest here, are the solutions based on AI because, due the improvement in this area in the last years, lots of new AI-based solutions for PdM have been proposed. Some examples are discussed in [42,43,44]. The adoption of AI-based solutions for predictive maintenance perfectly fits the vision of the fourth industrial revolution [45]. It is not a coincidence that all of the biggest IT companies started to propose their PdM solutions based on ML and cloud technologies: Amazon with their AWS Solutions [46], Microsoft with Azure [47], and Google with Google Cloud Platform [48].

### 3.2. Overview of Asset Administration Shell

The vision of Industry 4.0 encompasses a massive digitalization process where every asset from the physical world must be represented in the information world by a digital and uniquely-identifiable counterpart [12]. RAMI4.0 refers to such an entity with the name of AAS, but in the literature and other reference architectures like the Industrial Internet Reference Architecture (IIRA), it is referred to by the name DT. The AAS contains all the information relevant to a specific asset, its lifecycle, technical functionalities and it can also integrate procedures for the integration of sensor data and condition monitoring [49]. The conjunction of an asset with its relevant AAS defines the so-called I4.0-component [12], which refers to the concept of CPS in the context of RAMI4.0 [15]. In addition, it provides I4.0-compliant communication with the other I4.0 components in the value-chain network. Up to now, the only communication technology satisfying the requirements of Industry 4.0 is IEC 62541 (OPC UA) [50].

The concept of AAS is of paramount importance in the Industry 4.0 landscape because it is defined as the cornerstone of interoperability. Both the information model and the interface of AAS are standardized, coping with all the heterogeneous systems available in the industrial environment (industrial silos) [51]. Therefore, since AAS is an abstraction providing a common structure for the information relevant to assets and a common way to exchange such information [52], it enables the cooperation of assets based on different technologies.

#### 3.2.1. Structure of the AAS

**Figure 1:**
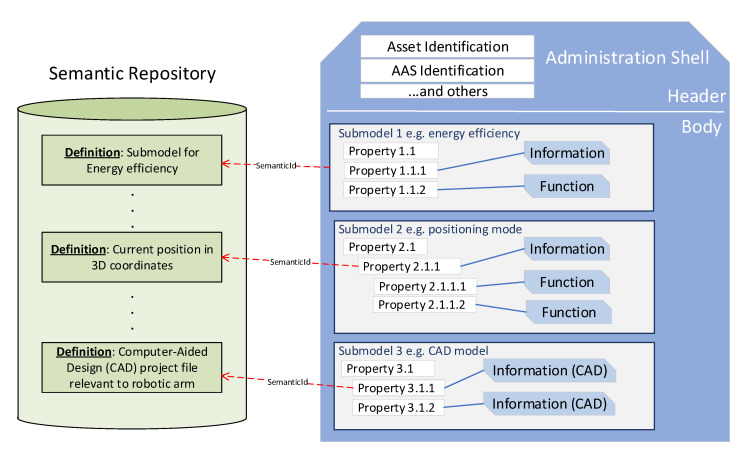
Structure of an Asset Administration Shell (AAS) from a high-level point of view.

From a high-level point of view, an AAS is structured as depicted in the right of Figure 1. It is composed by a header containing all the information regarding the identification of both AAS and asset, and a Body containing all the inherent information of the asset in the form of properties and functions (also named operations) [13]. Properties are defined as classified and mutually independent characteristics of systems that can be associated with values [53]. Functions, instead, are capabilities and actions that an asset performs. Both properties and functions are grouped in so-called submodels, each of which describes a specific aspect relevant to an asset (e.g., energy efficiency, positioning, documentation, drilling, maintenance, etc.). The aim is the standardization of a submodel for every aspect of an asset [54], but up to now, no standardized submodel has been provided, even though a lot of proposals have started to appear in the literature [17,26,55,56].

In the last few years, an AAS metamodel has been released to specify how the information of an AAS must be structured. In [14] the AAS metamodel is presented as a UML class diagram, defining all the “building blocks” that must be used to internally structure the AASs of every possible asset. Since the same entities of the metamodel may be used to define conceptually different elements (e.g., modelling both “height” and “rotation speed” as properties), such entities must be semantically annotated. Submodels, properties or functions composing an AAS contains a special attribute (semanticId) pointing to a semantic definition contained in an external semantic repository, as depicted in Figure 1. The term “semantic repository” is used here as a generic name identifying any sort of database or catalogue where all the semantic definitions reside, like the IEC Common Data Dictionary (CDD) [57] or eCl@ss [58]. In Figure 1, the semantic repository is represented as a single unit just from a logical point of view, since in practice a semantic repository is constituted by different databases or dictionaries located in different parts of the IT infrastructure.

One of the main features that an I4.0-component provides is “nestability” as discussed in [9]. The AAS of the composite component reflects the composition relationship referencing the ASSs of its components. The concept of composite component is deeply discussed in [59], and it is of paramount importance for the definition of the PdM model presented in this paper, as will be discussed in Section 4.

#### 3.2.2. Interfacing with an AAS

The AAS is a software entity providing its internal information to the external world by means of a standardized Application Programming Interface (API). Internal information, of course, is structured according to the AAS metamodel, as discussed previously. The API of an AAS is a CRUD-oriented interface (e.g., REST), thus data are accessible by a communication network so that an external client makes simple requests to the AAS to either retrieve or manage the data of interest. In general, RAMI 4.0 do not put any constraints about the location of an AAS: it may be embedded directly on a smart device or deployed in a completely different location (even though a connection with the asset may be maintained). Furthermore, it is worth noting that different parts of an AAS may be separated across the infrastructure, thus the AAS is not required, being a single monolithic software entity [12].

## 4. AAS-Based Model for Predictive Maintenance

In this section, the authors present a novel approach for the definition of a PdM solution in the context of a smart factory. Such approach is based on the considerations given so far and leverage mainly on the concept of the so-called logical block (LB). An LB is a modular element that groups functionalities relevant to a specific aspect of the PdM, like data acquisition or data manipulation. All the functionalities of an LB abstract a specific operation for the PdM process, regardless how such an operation is actually implemented. LBs and their functionalities are meant to be modular and cooperating elements, so that they can be used to describe a PdM solution (entirely or part of it) in a generic manner without considering implementation details. Describing PdM solutions in terms of generic functionalities permits to easily assign the right functionalities among the components of both IT and OT infrastructure. As will be discussed in the remainder of this section, describing a device in terms of its LB functionalities permits the definition of a role for that device. Such role identifies a sort of equivalence class between all the devices implementing the same functionalities. As a consequence, this makes the replacement of a device with an equivalent one seamless from the point of view of the PdM program.

The authors decided to use the concept of AAS and I4.0 Component for some of the device present in OT infrastructure to cope with the problem of heterogeneity of technologies present at that level, as discussed in the previous sections. The structure of the AAS fits for the realization of LBs and their functionalities, as will be discussed further. The common interfaces and the semantically enriched information provided by the AAS make this the foundation of the PdM models presented here. In fact, AAS achieves interoperability at the lowest level of the production infrastructure and thus allows a PdM program to be adapted to production reconfiguration. In the remainder of this paper, every device exposed by means of an AAS will be referred as an AAS-enabled device.

In the following subsections the PdM model will be presented following a bottom-up approach, describing it starting with the most fine-grained elements and ending with the high-level view of the model where all the interactions between components are highlighted.

### 4.1. Logical Blocks for PdM

Logical blocks are the most important elements of the AAS-based PdM model here presented as they abstract all the functionalities required for the PdM process. Such an abstraction generalizes and modularizes the description of the PdM solution and allows the implementation of same functionalities and operations using different technologies and approaches. For this reason, LBs can be realized following different standards or guidelines; for instance, functionalities for condition monitoring of machines may be based on standards like [25] or [60], but the model does not put any limitation on the standards or guidelines to be adopted for condition monitoring. AAS-enabled devices implement their LBs inside specific well-known submodels of their AAS. Such submodels will be described in the next subsection. For the sake of clarity, the term functionality here also means information, data structures and models, and not only operations.

**Figure 2:**
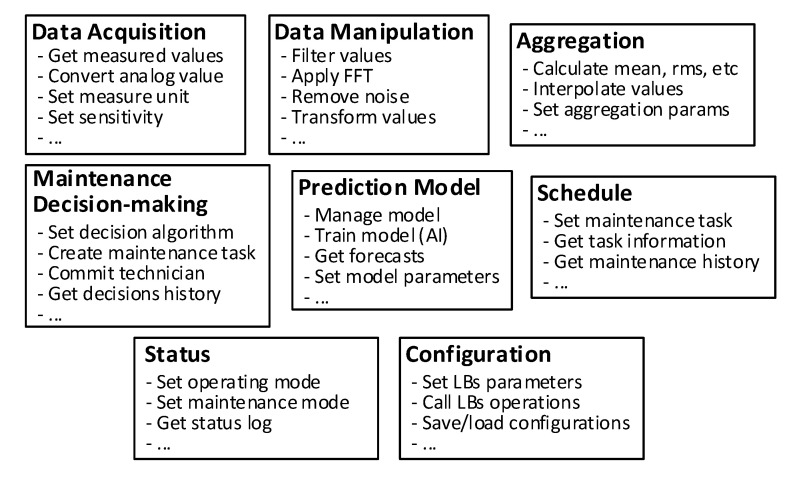
The logical blocks implementing generic functionalities related to predictive maintenance (PdM) aspects. FFT: fast Fourier transform; LB: logical block.

The LBs here presented have been defined considering the common aspects of the state of the art of PdM presented in Section 2 and Section 3.1. In Figure 2 some examples of functionalities are described for every LB.

In particular, the following LB descriptions identify the concepts discussed for the three main parts composing a general PdM solution, as described in Section 3.1:

- Data Acquisition (DA): the functionalities of this LB provide access to data coming from sensors. It involves functions like the conversion of the output of a transducer to a digital parameter representing the physical quantity. Such digital values may be enhanced with more quality parameters, like calibration or timestamp. Most of the approaches discussed in Section 3.1.1 fall within this LB.
- Data Manipulation (DM): this LB involves operations that perform analysis of signals and computes meaningful descriptors from raw measures (usually coming from DA). It also performs transformations on signals and applies algorithms for features extraction. Some of the approaches discussed for data processing in Section 3.1.2 are considered in this LB.
- Configuration (Config): this LB provides an interface for the configuration of other data-processing LBs exposing parameters and management functions. Some configurations for DA may include the relative position of the transducers, monitoring polling rates and calibration parameters, among others. Of course, parameters and functionalities of Config may be strictly dependent on the implementation of the other LBs. For instance, considering a block DA implemented using OPC UA and the subscription mechanism for data retrieval, a Config block may be used to configure parameters relevant to the publishing interval or sampling interval.
- Aggregation: this LB provides all the functionalities required for data aggregation of all the different data coming from logically “underlying” devices. Such a block may include mechanisms of sensor data fusion when, for example, the data monitored from a complex device come from sub-devices or sensors composing them. This perfectly fits with the concept of AAS because it allows the representation of complex devices by means of composition of the AASs of their sub-devices, as mentioned in Section 3.2.1. In these terms, for instance, the aggregation block implemented in the AAS of a complex device uses data coming from the DM or DA blocks in the AASs of the sub-devices. Furthermore, input data of an aggregation LB may come from other aggregation blocks, hence realizing an aggregation hierarchy, which is needed to manage the large amounts of data coming from sensors.
- Prediction Model: this LB identifies all the functionalities and facilities required for the diagnostics and prognostics of the monitored machinery. Most of the approaches described in Section 3.1.3 fall within this LB. For instance, considering a PdM solution based on artificial intelligence, this LB may consist of a neural network-based model or decision tree-model, but lots of different solutions may be adopted too. When it is possible, the models provided here are trained using historical data of both conditions and faults of machines. Such data are gathered and eventually manipulated by other LBs, discussed previously. Furthermore, the models may be constantly trained using data gathered in real time from AASs or forecast errors may be used to improve the accuracy of the model. The output of the prediciton model block depends on its implementation, and thus on the PdM technique adopted. Examples of output may be a type of failure, an indicator of the machine’s status or the RUL, as discussed in Section 3.1.3. It is worth noting that technical personnel working on data analysis and the tools they use are considered entities implementing functionalities of the prediction model LB.
- Maintenance Decision-Making: this LB involves the process of analysis of the information given by the prediction model block to schedule appropriate maintenance actions for the predicted faulty machine. Therefore, this block involves the facilities for the scheduling of maintenance tasks, the eventual commitment of available technicians for the maintenance, and it is in charge to change the operational state of the machine (i.e., changing the operational state from “working” to “maintenance”). All these kinds of operations change information in the proper AAS submodel of the relevant devices, similarly to the approach presented in [17]. In general, most of the functionality provided by a computer maintenance management system may be considered part of the maintenance decision-making block. It is worth noting that the output of this LB may be used as a feedback for the prediction model block to adjust the accuracy of the model adopted or check its correctness.
- Schedule: all the information relevant to the maintenance tasks are collected under this LB. Such information includes the date and the duration of the maintenance and the operator committed for the maintenance operation. This LB also includes the history of maintenance operations as it can give an estimation about the condition of the machine to consider or, in the worst case, whether a replacement with a new one occurred.
- Status: this LB contains all the information about the status of the machine. In particular, it highlights when the machine is in operating mode or in maintenance mode. This information may be useful to check the general status of the plant or to label eventual data still being collected from the machine even during a maintenance operation.

### 4.2. AAS Submodel Supporting PdM

An AAS-enabled device may implement all the functionalities of their LBs inside a specific submodel. The structure of a submodel allows the definition of the PdM functionalities in terms of properties and operations, both semantically annotated. As discussed in Section 3.2, the main idea in Industry 4.0 is having a standardized submodel definition for every relevant aspect of an asset but, up to now, no standardized submodel definition has been specified. The proposal of authors involves the definition of new AAS submodels containing well-known LB functionalities, so that some of the steps of the PdM process may be assigned to AAS-enabled devices in a common and standardized manner. Furthermore, since AAS allows for composition, functionalities in a submodel may be represented as composition of functionalities of the AASs of sub-devices or logically underlying devices. For instance, configuration functionalities of a high-level device may be represented as a composition of several configuration functionalities of underlying devices.

**Figure 3:**
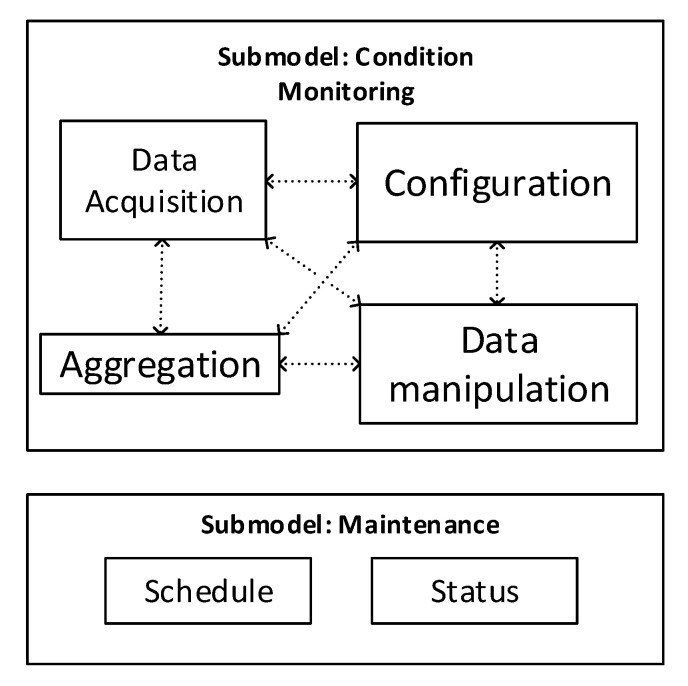
Submodel definition for condition monitoring and maintenance and the LBs they implement.

In this section, authors provide two possible definitions for submodels, specifying which LB they must implement. The two submodels defined here cover all the functionalities required for the condition monitoring of the machinery and the information relevant to the maintenance operations scheduled for a device; they are named “Condition Monitoring” and “Maintenance”, respectively. Figure 3 shows the LBs implemented internally by the two submodels, highlighting the relationships between them.

As shown in Figure 3, the submodel ”Condition Monitoring” implements the blocks DA, DM, Config and aggregation. The presence of the depicted LBs in the submodel is not mandatory. Whether an LB is implemented or not depends on the specific case being examined. For instance, a smart sensor may not implement the functionalities of the block aggregation inside its AAS. Instead, is quite common for a gateway implementing the functionalities of the LB aggregation in its AAS, whereas it is unusual implementing the functionalities of the block DA. All the LBs in the condition monitoring submodel may interact to each other, as depicted in Figure 3 by means of dotted arrows. Such interactions may represent data flows, events dispatching, function calls or parameter settings.

The submodel, maintenance implements, the functionalities of the block schedule and status. In general, the scope of this submodel involves everything concerning the maintenance tasks and operational condition of the device.

As the reader may have noticed, the blocks, prediction model and maintenance actions, are not considered inside the submodels’ definitions provided here. The authors decided to not include them because such high-level functionalities with high-demanding computational requirements may not be easily implemented in an AAS-enabled device. It is worth noting that this assumption is not a limitation; an AAS of a high-specialized tool may implement, if the solution requires so, the prediction model functionalities inside a well-known submodel.

### 4.3. Description of the PdM Model

The main aim of the proposed model is the harmonization of all possible approaches applied for PdM, which have been discussed in Section 3.1. This process has been realized with the definition of the LBs, grouping all the different aspects involved in a generic PdM solution, as mentioned in Section 4.1. Furthermore, as stated in the previous sections, the concept of AAS is used as the means to achieve the integration between different kinds of data coming from different devices and as an abstraction interface for all the heterogeneous operations that such devices implement (e.g., data retrieval, data aggregation, configuration, etc.). This is possible because the representation of the information and operations inside the AASs must follow the specification dictated by the AAS metamodel; therefore, different properties and functions are represented by means of the same well-known metamodel entities, as stated in Section 3.2.1. In Section 4.2, new submodel definitions for condition monitoring and maintenance have been defined, specifying the possible LBs, and thus PdM functionalities, that an AAS-enabled device may feature. Such submodels masquerade how the functionalities are implemented providing a uniform representation of their features to the external world. Furthermore, since the APIs provided by an AAS are standardized, they are the same for all the assets even though official specifications are still being defined and have not been released yet.

**Figure 4:**
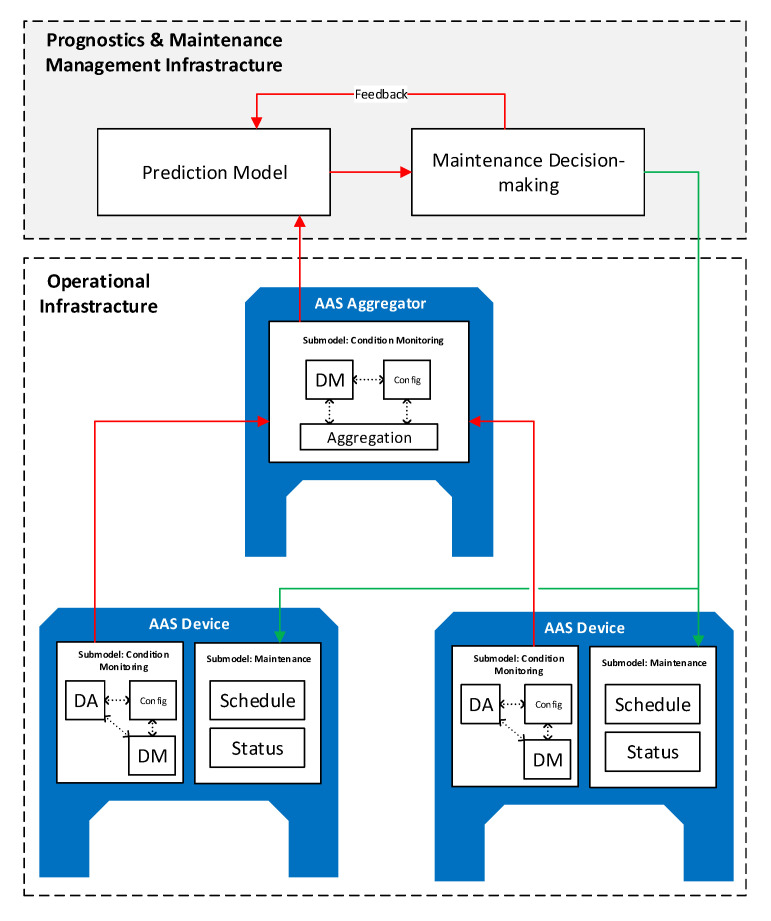
AAS-based PdM model for predictive maintenance. DM: data manipulation; DA: data acquisition.

From a high-level point of view, the PdM model is divided in two main parts: The operational infrastructure (OI) and the prognostics and maintenance management infrastructure (PMMI). The former encompasses all the elements of the plant involved in the PdM solution for the collection and manipulation of data to be used for maintenance prognostics. Examples of such elements are the machines to be maintained, industrial gateways, industrial PCs, but even high-level tools like mechatronic and embedded systems and applications (MES) and enterprise resource manufacturing (ERP) may be considered as being part of the OI. The latter, instead, encompasses all the elements of the PdM solution that use the data coming from the OI to forecast machine failures and schedule maintenance actions. Examples of such elements include AI models (e.g., recurrent neural network), tools for data analysis and software for the maintenance management. The structure of the PdM model is depicted in Figure 4, highlighting the relationship between components and the data streams from low-level devices to the PMMI elements for the decision-making task (red arrows). Furthermore, the figure shows how the topmost component interacts with the maintained devices to set their operational status and commit maintenance tasks (green arrows).

As shown in Figure 4, the OI consists of AAS-enabled devices providing both the data needed for the condition monitoring (e.g., production machinery that will require maintenance, sensors) and functions for data manipulation required from the first steps of the PdM process (see Section 3.1.2) (e.g., smart device, industrial PC, gateway). In general, what is considered as belonging to operational technology (OT) is part of the OI. Therefore, devices like sensors, actuators, machinery, but also Programmable Logic Controller (PLC), Supervisory Control And Data Acquisition (SCADA), Distributed Control System (DCS), may be considered part of OI. Information technology (IT) elements like databases, industrial PCs, or edge devices, like gateways, may be part of OI too. The presence of AAS is mandatory for the devices at the lowest levels of the infrastructure (i.e., brownfield) because, as mentioned in previous sections, such devices are the ones featuring a high degree of heterogeneity in the technologies and data representation adopted. Therefore, the AAS realizes the abstraction layer needed to achieve interoperability at that level. Of course, other elements of the IT infrastructure adopted in the PdM solution (like database or specific tools) may ideally have an AAS but this is not a strict requirement. The only requirement in the PdM model is, of course, that every PdM component that needs to interact with an AAS-enabled device must be able to communicate with the AAS API and understand its semantics.

PMMI is composed by entities whose nature is not defined in terms of device types but in terms of which functionalities they implement. In general, the functionalities implemented by the entities in PMMI involves, among others, the analysis of data coming from the OI, the development of a prediction model and a scheduler of task for maintenance actions (see Section 3.1.3). Contrary to the OI, the entities composing PMMI usually represent software entities or logical components rather than physical devices. Of course, databases and IT components exist at the PMMI level but since the presented model provides a functional viewpoint, what implements these functionalities is not pointed out. PMMI consists of IT elements and software components providing all the functionalities needed for data analysis, failures prediction, and scheduling of the maintenance tasks. The nature of such components is not specified but they are described only in terms of the functionalities they provide (i.e., their LBs). Such functionalities may be implemented in devices of the information technology (IT) infrastructure and/or in the cloud (in the case of a cloud-based PdM). For this reason, it makes no sense speaking about “devices” at this level since the only things that matter here are functionalities; how they are implemented strictly depends on the solution adopted for PdM. For instance, the prediction functionalities defined for PMMI may be implemented either by a recurrent neural network (artificial intelligence-based solution) or by a physical person consulting a visual tool for data analysis; even if the former is a software component and the latter is a person, both of them are considered entities of the PMMI. Furthermore, some facilities, like the ML tools, may be hosted in the cloud infrastructure of external companies (e.g., Microsoft Azure, Google Cloud platform), so there is no specific “physical device” to refer to in these cases. Since entities implementing functionalities in PMMI interact and use data coming from OI entities, it follows that they must understand the AAS API and data of the OI entities.

The AASs of the entities belonging to the OI are shown in Figure 4. The differentiation between device and aggregator, depicted in Figure 4, is not formal and is used just to clarify which role an entity plays in the OI. The role an entity plays depends on the LB it implements. For instance, the AAS device in Figure 4 represents a generic device that provides condition monitoring features and needs to be checked eventually for a maintenance task. Similarly, an AAS aggregator identifies a device that it is not a direct subject of the maintenance process, but an entity needed for it. In particular, the LBs it implements suggest that the role of the AAS aggregator is that of collecting all the data coming from different AAS devices and that does some sort of manipulation (e.g., data aggregation, sensor data fusion) before sending them to other entities. As already discussed, this entity categorization is not restrictive and new roles can be defined just by picking specific LB functionalities and combining them properly. As mentioned in Section 4.1, technicians may also be represented by means of LB functionalities and thus roles may be defined for them in accordance to their competences and their responsibilities in the PdM solution.

The role of an entity can be discriminated just looking at the LBs it implements. It is not required for an entity to implement all the functionalities described by one of its LBs. This aspect of the PdM model allows the definition of a sort of equivalence classes for PdM components because such roles are defined in terms of the collection of generic PdM functionalities. The advantage of the proposed model is describing a device using a role so that it can be replaced seamlessly with another device of the same role. All the PdM solutions may be represented according the AAS-based PdM model: all the relevant parts of the solution may be described in terms of generic LB functionalities, defining the roles that such PdM components play in the whole PdM solution. Generalization dictated by the model allows easy reconfiguration and extensibility of the production systems, increasing the integration of all the different parts of the PdM solution. In that sense, the AAS is the foundation of this abstraction mechanism for low-level devices featuring different implementations for similar functionalities, different information models or different communication protocols. The AASs make such devices I4.0 components, thus they can be easily replaced in a transparent fashion from the point of view of both the PdM solution and the production system. This aspect is highlighted in the case study described in the next section.

## 5. Case Study: PdM Solution for Milling Machines Using Cloud-Based Machine Learning

In this section a simplified version of a PdM solution based on machine learning for the maintenance of 100 milling machines will be described. The used case shows how the definition of an AAS-based model for PdM, defined in terms of LB, allows for the description of a PdM solution in a high-level perspective, specifying which functionalities and roles the components in the infrastructure have to play. As already mentioned in the previous sections, at the lowest level of the infrastructure this is possible only because the common standardized structure and communication interface of AAS hides the implementation details of the relevant asset and the technologies used to implement its functionalities. Therefore, generic LB functionalities can be implemented by an asset using any suitable solution because the AAS abstracts the implementation details and provides its information in a uniform (and semantically annotated) manner, exposed by a standardized API. For the sake of simplicity, the AAS mentioned in this case study is considered embedded in the relevant asset, which provides computation and communication capabilities (e.g., microprocessor and network access). This is not a limitation, since an AAS can be deployed in an IT component, other than its asset, provided that communication between the asset and the AAS is maintained, as stated in Section 3.2.2.

### 5.1. Description of the Case Study

The case study used here provides is a multi-class classification problem, thus, an ML algorithm is used to create the predictive model. Such model learns from the data collected from 100 milling machines, exposed by their AAS. The data used for the definition of the predictive model come from four different sources: real-time telemetry data from sensors (time-series), error logs, maintenance history, and machine information. Telemetry data require some manipulation to respect the desired information structure for the model training. Some manipulation may consist in simple measurement unit conversion or filtering, among others. Furthermore, the amount of telemetry data from each machine is aggregated every hour using an industrial PC. This first aggregation step is done averaging the data collected every hour. An edge gateway, instead, collects telemetry data from all the milling machines and labels the records accordingly with the relevant machine names (e.g., serial number or machine id). Finally, data are aggregated choosing a lag window of 24 h and using mean and standard deviation as rolling aggregate measures. All the records are then sent in the cloud where eventually other data manipulation processes may be applied to generate the data set for the training of the ML model.

Error logs are generated by milling machines collecting non-breaking errors that do not constitute failures. The error timestamps are rounded to the closest hour since telemetry data are aggregated at an hourly rate during the first aggregation step, as previously discussed.

Maintenance history is considered as a collection of records generated when a component is replaced during a scheduled inspection or replaced due to a failure. For the sake of simplicity, maintenance history is considered already available because collected in the past and stored in the databases.

Information about machine, instead, contains the model name of the milling machine and its age in terms of years of service.

### 5.2. Representing the Case Study in Terms of the PdM Model

Using the terms introduced by the model proposed in this paper, the AASs of the milling machines implements the LB DA and DM. The Industrial PC and the industrial gateway require the functionalities of the LB aggregation. All of them instead, require the functionalities of the LB configuration so that specific tools can interact with the AASs to set the right parameters for data acquisition, data manipulation and aggregation, respectively. As mentioned in the previous section, such functionalities and information must be implemented inside a “Condition Monitoring” Submodel”.

**Figure 5:**
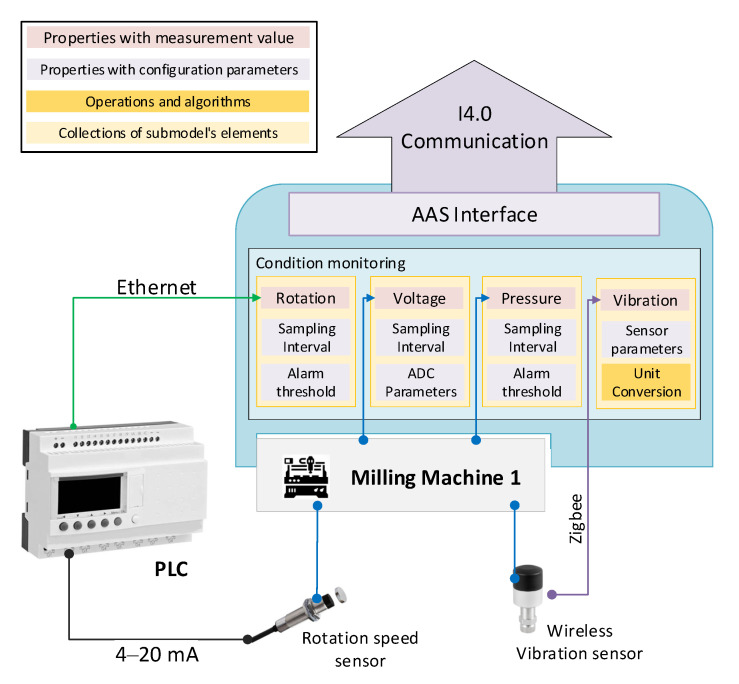
The structure of the AAS of a milling machine. The submodel “Condition Monitoring” provides measurement value, configuration parameters, alarms and operations in a uniform manner by means of the AAS interface. All the implementation details and underling technologies adopted for data collection and manipulation are hidden by the AAS.

The telemetry data are gathered using sensors directly applied to the milling machines or embedded in them. In particular, the data consists of vibration, voltage, rotation, and pressure. Voltage and pressure are gathered using sensors already embedded in the milling machine. The rotation speed is obtained using a rotation speed sensor applied to the machine and connected to a PLC. Finally, the vibration data are gathered using a wireless vibration sensor. All such different technologies and/or kind of data are hidden by the AAS of the milling machine and presented in a uniform manner inside the submodel “Condition Monitoring”, as shown in Figure 5.

The AAS of the milling machine organizes in its submodel “Condition monitoring” all the information relevant to the data of interest. Such information includes, among others, the measurement values, alarms thresholds, configuration parameters for data acquisition, and operations for data manipulation. The added value in the adoption of the AAS for such data exposure is its uniform and semantically annotated data structure. All properties and operations are modelled using the same entities coming from the AAS metamodel. As depicted in Figure 5, such uniform structure hides the complexity and the heterogeneity of the technologies required to gather data from the milling machine or to implement the operations. The measurement values rotation, voltage, pressure, and vibration are exposed by the AAS interface in a uniform manner, even though they are collected in different ways. For instance, the value of rotation is gathered using an ethernet-based protocol (e.g., Siemens Step7) to access the PLC internal memory containing the measured value, whilst the value of vibration is retrieved using a completely different communication protocol, i.e., Zigbee.

The AAS allows one to configure how such values must be retrieved (e.g., sampling rates) and manipulated. Ad-hoc configuration parameters and operations can be applied to data collected by the underlying sensors in order to expose values in a suitable manner for the ML solution of the use case. For example, let us consider the case of the vibration data, which usually are measured in m/s^2^ or g (gravitational constant equal to 9.81 m/s^2^). The value collected by the sensor is in millivolt but the AAS must expose such a value in m/s^2^. For this reason, the AAS contains some operations and algorithms that can be applied directly to data collected by the sensor to expose the value in a suitable manner. Thus, operation of the AAS obtains the value from the vibration sensor (measured in mV) and divides it for the sensitivity of the sensor (measured in mV/g) to convert it in terms of m/s^2^, as required by the ML algorithm of the case study. It is worth noting that the value of the sensitivity of the vibration sensor may be contained in a property of the AAS of the milling machine and used to parametrize the conversion operation. This is a simple case highlighting how the AAS abstracts the operations from the underlying implementation, i.e., the vibration sensor. Other operations implemented by the AAS may include, for instance, data filtering, fast Fourier transform (FFT), normalization, and so on. All of them may use other properties of the AAS to parametrize their applications.

**Figure 6:**
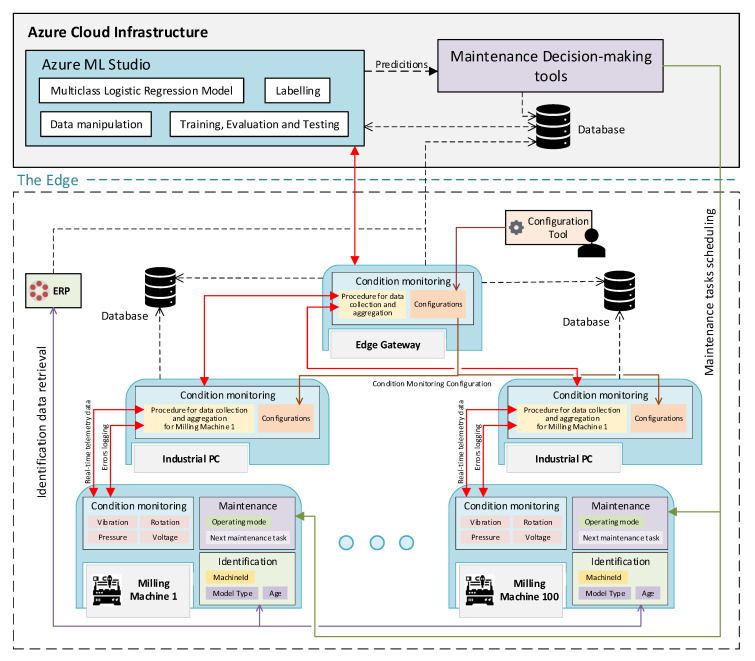
Complete overview of the use case scenario.

Figure 5 depicts only the information relevant to telemetry data, but the AAS of the milling machine exposes much more information. For instance, machine information like machineId, model name and age can be contained in an “Identification” submodel, as shown in Figure 6. This figure also shows the complete scenario of the case study and the interaction between all the components involved. Telemetry data exposed by the AASs of the milling machines are already well structured in a uniform manner. Therefore, the AASs of the industrial PCs are configured to collect measurement values from each relevant milling machine and saving them in a time-series database. Such data are then averaged in a lag window of an hour. Therefore, the AAS of the edge gateway is configured to collect the data of all the milling machines, putting them all together and calculating the mean and the standard deviation in a window of 24 h, as mentioned previously. Such records are than stored in a database in the cloud. How data collection, data manipulation, and aggregation is performed by the AASs is configured via a generic configuration tool that interacts with the functionalities contained in the sSubmodel “Configuration” of the relevant AASs. The configuration tool does not provide an AAS but knows how to interact with the AAS API of the edge gateway, as mentioned in Section 4.3. It is worth noting that the configuration of the edge gateway may start configuration processes to the underlying industrial PCs too. These cascading function calls are possible because of the inherent modularity of LB functionalities, realized in turn by the modularity of the structure of the AASs.

Some of the other information needed to form the dataset for the ML model can also be retrieved using the AASs. Error logs are generated by the industrial PCs collecting alarms coming from the AASs of the milling machines, whilst machine information can be directly retrieved by the Enterprise Resource Planning (ERP), which interacts with the AASs of the milling machines and save the information in the cloud, as depicted in Figure 6. As previously mentioned, maintenance history is already available in the cloud database.

In this use case scenario Microsoft Azure is used as the cloud infrastructure. Here, the tools offered by Azure ML Studio are used to prepare the dataset and train, test and validate the ML model for the failure predictions. The functionalities of the LB prediction model are obviously implemented by the tools of Azure ML Studio. Some functionalities of the LB Aggregation are also required at this level to put all data together and create the complete dataset.

With an AAS-based infrastructure under the edge, the collection of datasets of features is simplified since all the steps required for its construction are abstracted by LB functionalities. These are in turn implemented at the lowest levels in a standard and semantically annotated manner by the AASs. Furthermore, the interface exposed by the AAS hides the complexity behind the implementation of all such functionalities.

Once the ML-based prediction model is validated, real-time data can be provided to the model to make predictions, which implements, as already discussed, the functionalities of the LB prediction model. Real-time data can be collected (and eventually aggregated) using the same AAS-based infrastructure used to construct the dataset for the model training. The predictions are used by a maintenance decision-making tool deployed in the cloud that analyzes the inputs and eventually schedules maintenance actions. This tool interacts directly with the AASs to set all the maintenance information in the properties of the submodel “Maintenance”. The latter implement the functionalities required by the LB schedule and status, which exposes information relevant to the maintenance actions and to the operational state of the milling machine, respectively. Technicians committed to the task can then interact with the AAS of the milling machine to retrieve additional information about the maintenance action.

This case study shows how the definition of a maintenance solution in terms of LB functionalities abstracts the approach adopted for the PdM. Furthermore, using LBs helps to identify the roles that IT elements must play in order to realize the PdM program. The adoption of an AAS-based model is of paramount importance in this context because the implementation of LBs can be hidden by the standardized structure and interface of the AAS. As we have seen, this is a huge improvement in terms of interoperability, especially in the brownfield, where lots of different vendor-specific technologies can be used for data retrieval and for communication. For the LBs prediction model and maintenance decision-making instead the functionalities can be directly implemented using vendor specific tools (like Microsoft Azure in our case), but since these LBs define high-level functionalities, changing the PdM solution (e.g., different ML algorithm or model) or the cloud platform adopted (e.g., Google Cloud Platform) is not as constraining as the process of gathering and manipulating the flow of data from several different sources. The harmonization of the technologies in the OT level using the AASs, as proposed by the authors, is an evident advantage of the definition (or re-definition) of a PdM solution in the context of Industry 4.0.

## 6. Conclusions and Outlook

This paper assesses the state of the art PdM and generalizes the steps and the functionalities needed for the definition of a model able to describe every PdM solution in terms of a combination of generic functionalities. This is of paramount importance in the context of Industry 4.0 and smart manufacturing, where production systems adapt their configuration on the basis of the enterprise needs; replacing machinery or adding new devices in the production configuration may harm the PdM program. To adapt a predictive maintenance solution to production re-configuration there are two main requirements: a model providing functionalities for the description of a technology-independent PdM solution and an abstraction mechanism for all the different technologies adopted for the PdM implementation. The related works do not address both requirements together, hence the proposed PdM model defines a new approach to satisfy both. The advantages of the proposed PdM model rely on both the concept of LB and AAS. The former is a conceptual group of functionalities related to the same aspect of a PdM solution and allows the definition of roles for the components of the maintenance program, which enable both easy replacement and alterations in the PdM solution in a seamless manner. The latter, instead, provides an abstraction layer for the heterogeneity of devices and technologies adopted (especially in the brownfield), improving the degree of integration between PdM components and a common structure to the information and operation featured by devices. Furthermore, the structure of the AAS perfectly fits the realization of LB functionalities using semantically annotated properties and functions inside their submodels. Both the LBs and AASs adopted in the PdM model presented here allow the definition of maintenance programs to improve the level of flexibility in production. A cloud-based PdM program has been presented as a case study scenario where the AAS-based PdM model is used to define the steps in terms of LB functionalities. Furthermore, the case study highlights how the AAS is a powerful Industry 4.0 concept that hides the implementation details behind PdM solutions, increasing the interoperability between both devices of different manufacturers and devices using different information models.

Since the AAS contains all the information relevant to an asset (organized in proper submodels), future work can demonstrate how new information generated by procedures of different areas (like PdM) can be used to improve production flexibility. For instance, a manufacturing system may access the AAS of a machine to retrieve its operational status; if the machine is either in maintenance or in failure mode, the manufacturing system can use such information to assign the next production step to a functional equivalent machine. This may show how the results coming from the PdM solution may be used to improve the availability and the optimization of the production system.
